# Using AlphaFold Predictions in Viral Research

**DOI:** 10.3390/cimb45040240

**Published:** 2023-04-21

**Authors:** Daria Gutnik, Peter Evseev, Konstantin Miroshnikov, Mikhail Shneider

**Affiliations:** 1 Limnological Institute of the Siberian Branch of the Russian Academy of Sciences, 3 Ulan-Batorskaya Str., 664033 Irkutsk, Russia; daria_gutnik@mail.ru; 2 Shemyakin-Ovchinnikov Institute of Bioorganic Chemistry of the Russian Academy of Sciences, 16/10 Miklukho-Maklaya Str., GSP-7, 117997 Moscow, Russia; petevseev@gmail.com (P.E.); mm_shn@mail.ru (M.S.)

**Keywords:** AlphaFold review, viruses, bacteriophages

## Abstract

Elucidation of the tertiary structure of proteins is an important task for biological and medical studies. AlphaFold, a modern deep-learning algorithm, enables the prediction of protein structure to a high level of accuracy. It has been applied in numerous studies in various areas of biology and medicine. Viruses are biological entities infecting eukaryotic and procaryotic organisms. They can pose a danger for humans and economically significant animals and plants, but they can also be useful for biological control, suppressing populations of pests and pathogens. AlphaFold can be used for studies of molecular mechanisms of viral infection to facilitate several activities, including drug design. Computational prediction and analysis of the structure of bacteriophage receptor-binding proteins can contribute to more efficient phage therapy. In addition, AlphaFold predictions can be used for the discovery of enzymes of bacteriophage origin that are able to degrade the cell wall of bacterial pathogens. The use of AlphaFold can assist fundamental viral research, including evolutionary studies. The ongoing development and improvement of AlphaFold can ensure that its contribution to the study of viral proteins will be significant in the future.

## 1. Introduction

**Figure 1:**
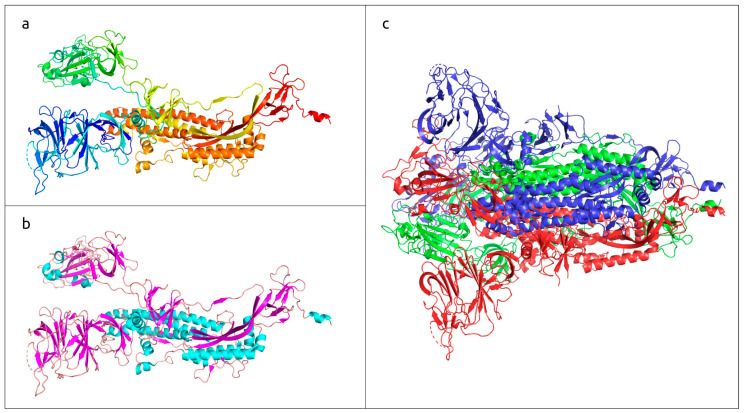
Three-dimensional structure of SARS-CoV-2 trimeric spike glycoprotein, determined with electron microscopy (PDB code #7DF3 [7]). (**a**) Monomeric subunit coloured based on a rainbow gradient scheme, where the N-terminus of the polypeptide chain is coloured blue, and the C-terminus is coloured red. (**b**) Monomeric subunit coloured based on the secondary structure, where α-helices are coloured cyan, β-sheets are coloured magenta, and loops are coloured wheat. (**c**) Quaternary structure of functional trimer, where each monomer is coloured in a different colour.

Proteins play a crucial role both in building biological structures and in managing biochemical processes in living organisms. Proteins are linear unbranched polymers of amino acid residues. To possess biological activity, proteins adopt unique three-dimensional structures (folds), which is known as the “native state” [1,2]. The folded structure is determined by the amino acid sequence of the protein (“primary structure”) [3,4], and the formation of the folded native conformation (“tertiary structure”) starts with rapid folding into a “secondary structure”, which is a local spatial conformation of the polypeptide backbone, stabilised by intramolecular hydrogen bonds [5]. The most common elements of the secondary structure are α-helices and β-sheets. The so-called “quaternary structure” is the result of assembly of the folded proteins or protein subunits into protein complexes of fully functional protein [6]. Thus, the protein structure can be described using four levels of organisation: a primary, secondary, tertiary and, for some proteins, quaternary structure (Figure 1).

Knowledge of the three-dimensional structure of proteins is important for understanding their functions. A detailed knowledge of three-dimensional structure is crucial for protein structure-based drug design [8]. The main techniques for determining protein structures are X-ray crystallography [9], NMR spectroscopy [10] and Cryoelectron microscopy [11]. Experimentally determined protein structures are stored in databases, the largest of them being the publicly available Protein Data Bank (PDB) (https://www.rcsb.org/, accessed on 1 March 2023). As of March 2023, the PDB database contained about 202,000 experimentally determined structures, most of which belonged to proteins. This is, however, just a small fraction of all proteins for which the primary sequences are known. The UniProtKB/TrEMBL database alone contains over 200 million sequence records, (database release 2022_05 of 14 December 2022 contained 229,580,745 sequence entries, https://www.ebi.ac.uk/uniprot/TrEMBLstats, accessed on 1 March 2023). Thus, the prediction of the three-dimensional structure of a protein is an urgent problem that aims to fill the gap between the large known number of primary sequences and the relatively small number of known structures.

Prediction of the three-dimensional structure of proteins is a difficult task. For a long time, the main prediction methods included comparative modelling (homology modelling), threading and ab initio and machine-learning approaches [12,13]. The development of end-to-end machine-learning approaches in recent years has resulted in the emergence of new techniques that can often outperform other methods [2,14]. Moreover, recent progress associated with deep-learning methods enables speculation about a revolution in protein-structure prediction [15]. One of the most popular deep-learning techniques is Alphabet–Google DeepMind’s neural network-based end-to-end solution AlphaFold2 (AlphaFold, AF2), which was presented in the CASP14 competition [16], the second iteration of the AlphaFold system entered in CASP13 [17]. AlphaFold employs a deep-learning approach and a conventional neural network. This technique is able to predict the distance and torsion distribution of proteins, using training schemes of experimentally determined PDB structures, protein primary sequences and the multiple sequence alignment (MSA) of proteins. In CASP14, AlphaFold2 structures had a median backbone accuracy of 0.96 Å RMSD_95_ (Cα root-mean-square deviation at 95% residue coverage) and an all-atom accuracy of 1.5 Å RMSD_95_. The corresponding values for the prediction of the best alternative method were 2.8 Å and 3.5 Å [16]. The high level of accuracy of AlphaFold2 predictions boosted the popularity of this technique. One might even talk about “AlphaFold mania”, given the astonishing increase in the number of journal articles and preprints citing AlphaFold2 AI software [18]. As of the beginning of March 2023, the original paper [16] published in July 2021, which described AlphaFold2′s release, with its source code, was accessed about a million times and, according to the Web of Science metric, was cited about 5000 times (https://www.nature.com/articles/s41586-021-03819-2/metrics, accessed on 1 March 2023).

The updated version of AlphaFold2, called AlphaFold-Multimer, also developed by DeepMind, was released several months after AlphaFold2 [19]. AlphaFold-Multimer was designed to predict the three-dimensional structure of protein complexes. AlphaFold-Multimer was benchmarked on a large dataset of 4446 protein complexes, successfully predicting the interface in 70% of cases of heteromeric interfaces and in 72% of cases of homomeric interfaces. A high level of predictive accuracy was demonstrated in 26% of cases of heteromeric interfaces and 36% of cases of homomeric interfaces.

The level of accuracy of AlphaFold (and other AI protein-folding methods, such as RoseTTAFold [20]) makes it tempting to use AlphaFold predictions in various fields of biological and medical research. In particular, virology, the importance of which has become especially evident in the light of the recent COVID-19 pandemic, has received a new tool that can solve a number of problems requiring the knowledge of three-dimensional protein structures. Virology studies viruses, probably the most widespread entities on Earth [21]. Viruses infect various cellular organisms, including eukaryotes, archaea and bacteria. In the latter case, they are called “bacteriophages”, or “phages”. Phages and their proteins that are harmful to bacteria can be used to fight bacterial infection in humans, animals and plants [22,23]. So-called “phage therapy”, or the use of bacteriophages to treat bacterial infections, can assist in the context of the rise of antimicrobial resistance [24]. This review describes different cases of the use of AlphaFold for the purposes of viral research. It summarizes the results of the studies involving AlphaFold predictions, analyses the possible advantages and disadvantages of AlphaFold for predictions of viral proteins and discusses corresponding studies (Table 1).

## 2. Application of AF2 for Research on Eukaryotic Viruses

### 2.1. Application of AlphaFold for SARS-CoV-2 Research

The outbreak of severe acute respiratory syndrome caused by coronavirus 2 (SARS-CoV-2, realm *Ribozyviria*, class *Pisoniviricetes*, order *Nidovirales*, family *Coronaviridae*, genus *Betacoronavirus*) and the spread of associated infection boosted research on coronaviruses. The structure of SARS-CoV-2 spike (S) glycoprotein, the main target of antibodies, has been determined by cryo-electron microscopy and was used in the development of vaccines and inhibitors [82,83]. S glycoprotein promotes entry into the cell. Another target of drug design is main protease cutting the initial translated propeptide into functional viral proteins. The crystal structure of the SARS-CoV-2 main protease was also obtained experimentally [84].

To assist the solution of tasks related to general research and drug design, different structure prediction techniques, including AlphaFold, were used for prediction of SARS-CoV-2 proteins [25,26,27,28,29,85]. The main task was probably the investigation of the mechanism of interaction of the SARS-CoV-2 receptor-binding protein (RBP), which is the SARS-CoV-2 spike, and the angiotensin-converting enzyme 2 (ACE2) receptor. AF2 predictions enabled clarification of the structural features of monomeric and multimeric formulations of the vaccine and suggested that monomeric formulation presents more antigenic epitopes [27]. The emergence of new immune-escaping variants of SARS-CoV-2, such as Omicron BA1, made it important to study potential mutation sites that do not yet exist in nature but could increase the binding affinity of RBD and the receptor [29]. AF2 predictions were successfully used to find an explanation for the observed reduction in the neutralisation of SARS-CoV-2 variants of concern compared with other variants [28]. AF2 predictions can be combined with molecular dynamics simulations to improve modelling accuracy [86] and to predict the physical properties of proteins. Such models can be used for studies of both qualitative and quantitative aspects of the formation of the quaternary structure of proteins [85]. AlphaFold models are useful for revealing possible ligand binding sites. Together with virtual screening and in silico validation, these approaches provide the basis for the biological testing of new drugs and for the repurposing of natural products [25].

**Figure 2:**
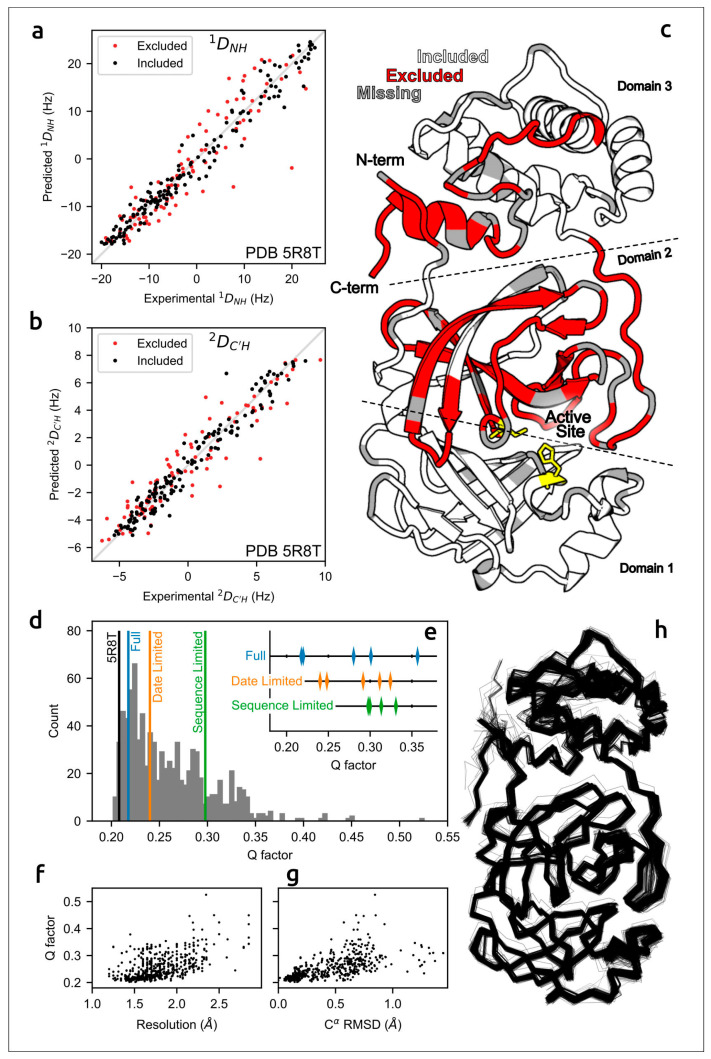
Agreement between measured M^pro^ RDCs and values predicted by AF2-derived models. (**a**) ^1^*D*_NH_ and (**b**) ^2^*D*_C′H_ experimental couplings vs. those predicted from X-ray structure 5R8T. (**c**) Excluded residues (red) illustrated on a ribbon diagram (PDB code 5R8T; only a single chain is shown, for clarity); residues with missing RDCs are shown in grey and the catalytic dyad is shown in yellow. (**d**) *Q*-factors from SVD fits of ^1^*D*_NH_ and ^2^*D*_C′H_ RDCs to the *included* region of all available M^pro^ X-ray structures, plotted as a histogram, with the top-ranked (Amber-relaxed) AF2 models obtained using *full*, *date-limited* and *sequence-limited* implementations marked. (**e**) *Q*-factors of all Amber-relaxed models. (**f**) X-ray structure resolution vs. *Q*-factor and (**g**) C^α^ RMSD (relative to 5R8T) vs. *Q*-factor. (**h**) C^α^ wireframe of all 352 PDB structures. Images courtesy of Dr. Adriaan Bax. Reprinted/adapted with permission from Ref. [30]. Not subject to U.S. Copyright.

The accuracy of predicted structures can be assessed using computational techniques [87] and via experimental methods, e.g., optical spectroscopy or measurement of solution residual dipolar couplings data (RDCs) [30,88]. A meticulous evaluation of the concordance of AF2 models of the SARS-CoV-2 homodimeric 3C-like protease (M^pro^) with residual dipolar couplings (RDCs) measured in solution for ^15^N–^1^H^N^ and ^13^C′–^1^H^N^ atom pairs indicated the close agreement of AlphaFold predictions with experimental data (Figure 2) [30].

Interestingly, the high level of accuracy of AF2 predictions makes it possible to use AlphaFold predictions to determine a macromolecular structure from crystallographic diffraction experiments. It has been shown that a template-free AF2 model, generated by the AlphaFold2 group, was of sufficient quality to phase the native SARS-CoV-2 ORF8 dataset by molecular replacement, overcoming the limitations of the crystallographic phasing problem [26]. However, a comparison of RMSD (root mean square deviation of atomic positions) values of SARS-CoV-2 spike RBD, the laboratory-derived structure with both trRosetta-generated models [89] and models generated by AlphaFold v2.1.0, indicated the high level of accuracy of both methods, but the better results were obtained with trRosetta.

### 2.2. Application of AlphaFold to Study Eukaryotic Viruses

AlphaFold is widely used in research on other eukaryotic viruses, including monkeypox virus (MPXV) [31,32,33,34], herpes simplex virus [35,36], hepatitis E virus (HEV) [37] and other viral pathogens of humans and economically significant animals and plants [38,39,40,41,42,43]. Monkeypox virus (MPXV) represents a new serious threat to human health. MPXV has spread to 110 countries (https://www.cdc.gov/poxvirus/mpox/response/2022/world-map.html, accessed on 1 March 2023). As of 1 March 2023, there were 86,231 confirmed cases worldwide, of which 84,858 cases occurred in locations that had not previously reported MPXV cases. Monkeypox virus is classified as a member of realm *Varidnaviria*, class *Pokkesviricetes*, order *Chitovirales*, family *Poxviridae*, genus *Orthopoxvirus* and is evolutionarily close to vaccinia virus (VACV), the smallpox virus. AlphaFold-derived structures of the recombinantly expressed MPXV antigen truncations to their VACV homologues have indicated that MPXV and VACV antigens are likely to achieve similar conformations [34]. The World Health Organisation (WHO) has recommended the current anti-smallpox drugs tecovirimat, brincidofovir and cidofovir for the treatment of monkeypox [90]. Brincidofovir and cidofovir inhibit DNA polymerase (DNAP), while tecovirimat is an inhibitor for poxvirus phospholipase D (protein F13) [91], but specific antiviral treatment requires new drugs.

MPXV DNA polymerase (DNAP) is a very important antiviral drug target. The laboratory-derived structure of MPXV DNAP was deposited in the RCSB PDB database (PDB code 8HG1) in mid-November 2022, and a paper describing this structure was published in January 2023 [92]. Before that, the AF2-derived structure was obtained and used in the search and design of new inhibitors of MPXV DNAP. The molecules found were predicted to bind to the MPXV DNAP with a binding energy comparable to that of brincidofovir and cidofovir. New MPXV DNAP inhibitors are important in the context of possible drug resistance, which can arise due to mutations in proteins of the DNA replication complex (RC). Studies of the effect of mutations in MPXV RC using AF2-generated models have suggested similar mechanisms of drug resistance to cidofovir in monkeypox and vaccinia viruses [32]. It appears that the use of highly accurate AlphaFold predictions can assist the forecasting of the emergence of drug-resistant variants of concern to improve preparedness for them.

**Figure 3:**
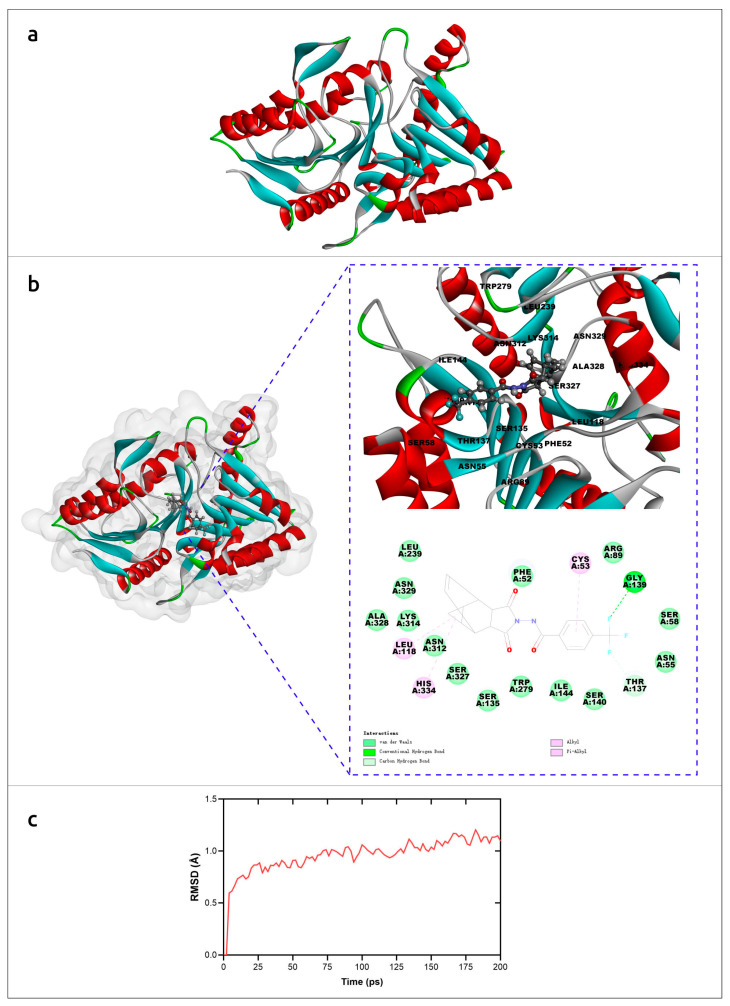
Molecular simulation analysis of tecovirimat with F13 from monkeypox virus. (**a**) Overview of the F13 protein structure from monkeypox virus generated by AlphaFold. (**b**) The minimum free energy poses with of F13 protein and tecovirimat and corresponding interactions plots. (**c**) RMSD of monkeypox virus F13-tecovirimat complex during the production stage of molecular dynamics. Images courtesy of Dr. Leiliang Zhang. Reprinted/adapted with permission from Ref. [33]. © 2022 The British Infection Association.

The molecular mechanism of interaction of tecovirimat with the monkeypox phospholipase D (F13) was studied using AlphaFold models and molecular dynamics simulations [33]. The results suggested a detailed mechanism of inhibition of F13 by tecovirimat (Figure 3) and supported the efficacy of tecovirimat against monkeypox virus, emphasising the importance of the availability of precise modelling for revealing molecular mechanisms of drug action.

The development of new drugs is barely possible without an understanding of the mechanisms of viral infection. This knowledge can often require robust structural analysis, which can make use of modern deep-learning structure prediction methods. AlphaFold can facilitate the elucidation of the functionality of viral proteins.

Herpesviruses constitute an important group of pathogens that infect animals, including humans. Herpesviruses infect most vertebrates, causing a lifelong latent infection [93]. Herpesviruses belong to the realm *Duplodnaviria*, class *Herviviricetes*, order *Herpesvirales*, and comprise the families *Alloherpesviridae*, *Herpesviridae* and *Malacoherpesviridae* [94]. Human herpesviruses belong to the family *Herpesviridae*. Herpes simplex virus 1 (HSV-1) (genus *Alphaherpesviruse*), residing in sensory neurons or sympathetic neurons, has been shown to severely modify infected cells and to remodel the composition and architecture of cellular membranes [35,95,96]. One of the HSV-1 proteins, phosphatase adaptor UL21, mediates dephosphorylation and accelerates the rate of ceramide to sphingomyelin conversion, altering cell membranes and influencing viral replication [35]. AlphaFold-Multimer modelling has revealed the details of the interaction of UL21 and viral protein UL16 and has enabled the suggestion of the functionality of domains of the latter protein using its structural features. Specific protein–protein interactions have been shown to be essential for lipid metabolism [35]. The use of AlphaFold has also shown that another HSV-1 protein, the tegument protein UL37, interacts with the cytoplasmic surface of the lipid membrane, suggesting that UL37 can be a peripheral membrane protein [36]. AlphaFold predictions have suggested the domain organisation of UL37, and assisted experimental studies and molecular dynamics simulation have clarified the structural features and molecular mechanisms of UL37 interactions.

Fundamentally similar tasks concerning research on other viral pathogens of animals, including humans, and plants can be made easier by the use of AlphaFold predictions. These tasks include mechanisms that are crucial for viral attachment, penetration, replication, release and other steps in the viral infection cycle. They can include the investigation of viral proteins and membranes [38,41,43], viral proteins and DNA [39] and studies of viral proteins, glycoproteins and their mutations [37,40,42]. It is noteworthy that AlphaFold predictions are often used as part of an integrated approach, making the planning of experiments easier and improving understanding of the results obtained.

## 3. Application of AlphaFold for Research on Bacteriophages

Bacteriophages (a.k.a. phages) are viruses that infect and replicate in bacterial cells alone. Bacteriophages are ubiquitous—they can be found in water, soil and various living organisms [97]. The total number of bacteriophages can be estimated at 10^31^ viral particles, which is 10–100 times the number of cells [98]. The total mass of these particles is about a trillion tons [99]. Phages are also members of plant and animal microbiomes, including humans. For example, the human gastrointestinal tract contains more than 10^12^ phage virions [100]. The ability of bacteriophages to destroy the cells of pathogenic bacteria attracted the attention of scientists as early as the beginning of the 20th century. In recent decades, interest in bacteriophage therapy has begun to grow, primarily due to the spread of antibiotic resistance. Phage therapy has important advantages [101], including sustained bactericidal activity and “autodosing”, wherein the number of phages positively correlates with the number of host bacteria. Furthermore, phages have low intrinsic toxicity, and phage therapy is characterised by minimal disruption of normal flora and the lack of cross-resistance with antibiotics.

**Figure 4:**
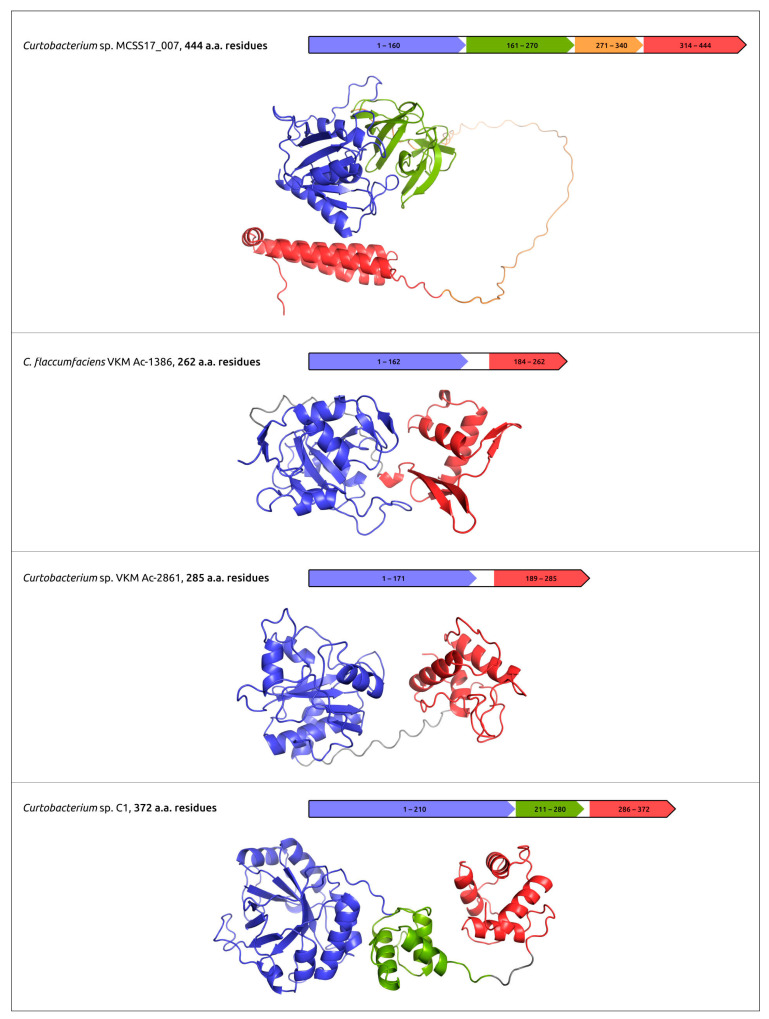
Predicted domain architecture and AlphaFold models of putative endolysins encoded in the prophage-derived regions. Reprinted/adapted with permission from Ref. [47]. © 2023 by the authors.

The practical use of phages for phage therapy requires an understanding of the structural bases of interactions of the host receptor and phage receptor-binding proteins (RBPs); the latter can include tail fibre and tail spike proteins (TFP and TSP). In addition, phage RBPs, as well as endolysins and ectolysins, the proteins that cause cell lysis, can be used as antibacterial agents by themselves [45,102]. The analysis of the structural features of phage RBPs and lysins can use modern deep-learning techniques, including AlphaFold. Together with experimental studies, AlphaFold predictions can be used to elucidate the domain organisation of TFP, TSP and cell-wall degrading enzymes, to reveal the sites of phage particle binding and enzymatic domains (Figure 4) [45,46,47,52].

As well as in the case of eukaryotic viruses mentioned above, AlphaFold predictions can contribute to building the model of the viral particle [48,103] or the virion parts, including the attachment apparatus [46,50] and phage egress machinery [51]. All the steps of phage infection are accompanied by macromolecular interactions that include proteins, so AlphaFold’s highly accurate structural predictions can assist in the elucidation of the mechanisms of the formation of the phage nucleus [49], lysogeny maintenance [53] or anti-phage defence [44,54]. AlphaFold can also be useful in the trivial but relevant task of phage genome annotation, assisting the prediction of genes’ functionality. As of January 2023, 19,499 GenBank sequences, assigned to class *Caudoviricetes*, contained 1,731,815 coding regions, 67% of which were annotated as hypothetical proteins. In some cases, BLAST search and HMM-HMM motif comparisons fail to assign a function to proteins encoded in phage genomes, but analysis of fold of AF2-derived structures can assist to clarify this function [55].

**Figure 5:**
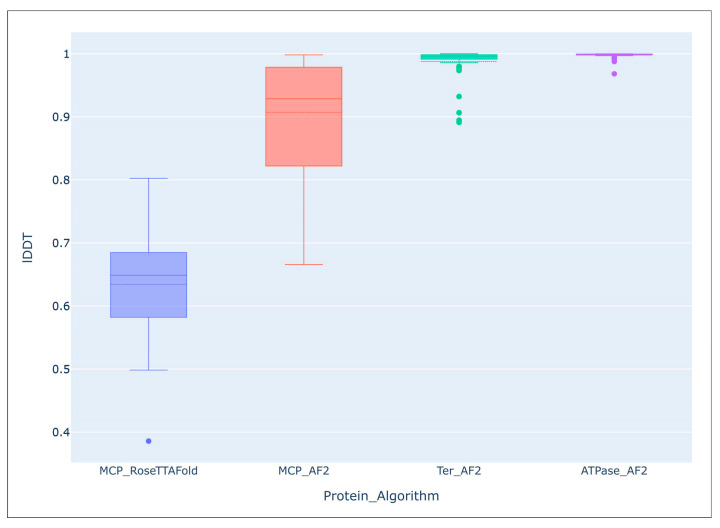
Comparison of the overall accuracy of predictions made with the Local Distance Difference Test (lDDT), using the DeepAccNet accuracy predictor. MCP_RoseTTAFlold–RoseTTAFlold models of the MCP, MCP_AF2–AlphaFold models of the MCP, Ter_AF2–terminase ATPase subunits’ models predicted with AlphaFold, ATPase_AF2–ATPase domain of terminase ATPase subunits’ models predicted with AlphaFold. Reprinted/adapted with permission from Ref. [55]. © 2023 by the authors.

It seems that no large-scale studies have been published on the accuracy of modelling using AF2 compared with the predictions of other algorithms. However, comparing the predicted average local distance difference test (lDDT) score of the 54 AF2-derived models of the major capsid protein and ATPase subunit of phage terminase indicated an impressive level of accuracy of the predictions [55]. Interestingly, structural predictions of more conserved terminase were more accurate than those of major capsid protein, (terminase lDDT mean: 0.988, median: 0.996; major capsid protein lDDT mean: 0.907, median: 0.929). The average lDDT of the ATPase domains extracted from the ATPase subunit of phage terminase models was even higher (mean: 0.998, median: 0.999). An evaluation of models of the same major capsid proteins, carried out using a different deep-learning algorithm, RoseTTAFold, showed a lower accuracy of prediction (lDDT mean: 0.634, median: 0.649) than with the AlphaFold models (Figure 5).

## 4. Application of AlphaFold for Evolutionary and Taxonomic Studies

**Figure 6:**
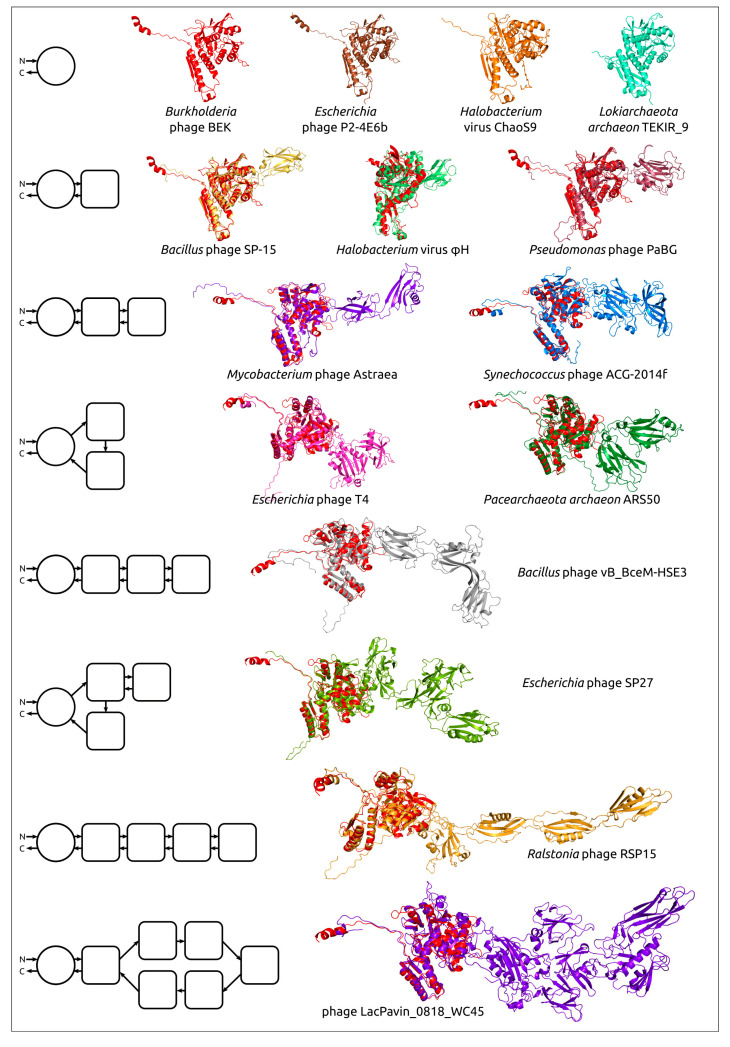
Examples of the structural architecture of AF2-derived contractile phage sheath proteins [58]. Proteins consisting of two and more domains are superimposed with the modelled structure of the *Burkholderia* phage BEK tail sheath protein, depicted in the red colour. The schemes on the left show the structural architecture of proteins. The main domain is depicted as a circle, with additional domains represented as squares with rounded corners. The direction of the polypeptide chain, from the N- to the C-termini, is shown with arrows. Reprinted/adapted with permission from Ref. [58]. © 2022 by the authors.

Comparing structural similarity and specific structural features can clarify the evolutionary relationships between proteins. Furthermore, the emergence of new high-precision algorithms for predicting the structure of proteins, including AlphaFold, can enable the identification of evolutionary relationships between highly divergent discovered proteins, using the results of structural modelling. The evolution of proteins may be accompanied by the appearance of new domains, and comparative analysis of AF2-derived structures can help reveal patterns of protein evolution. Studies of bacteriophage tail sheath proteins, an important part of phages’ contractile injection system, have enabled the identification of the common core domain, including both N-terminal and C-terminal parts. The remaining variable parts consisting of one or more moderately conserved domains have, presumably, been added during phage evolution (Figure 6) [58].

**Figure 7:**
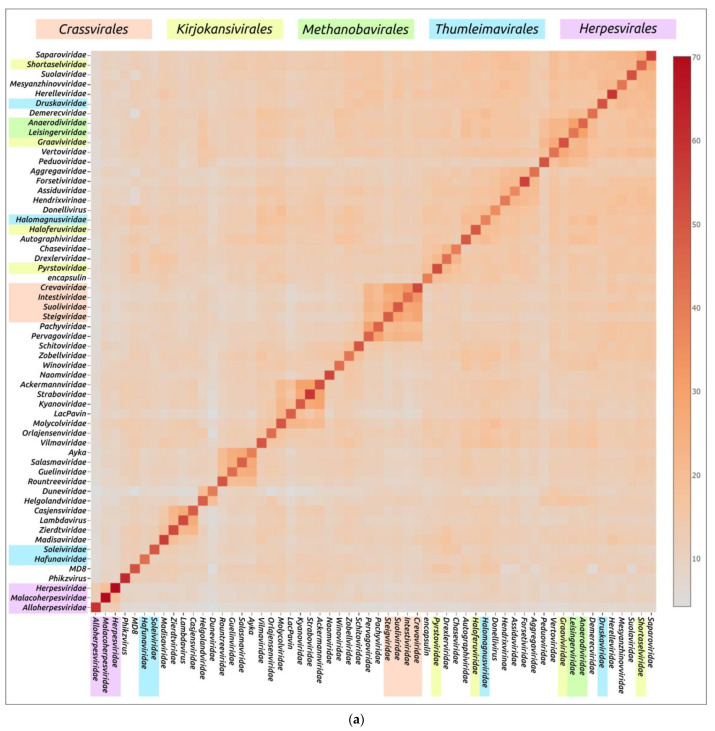

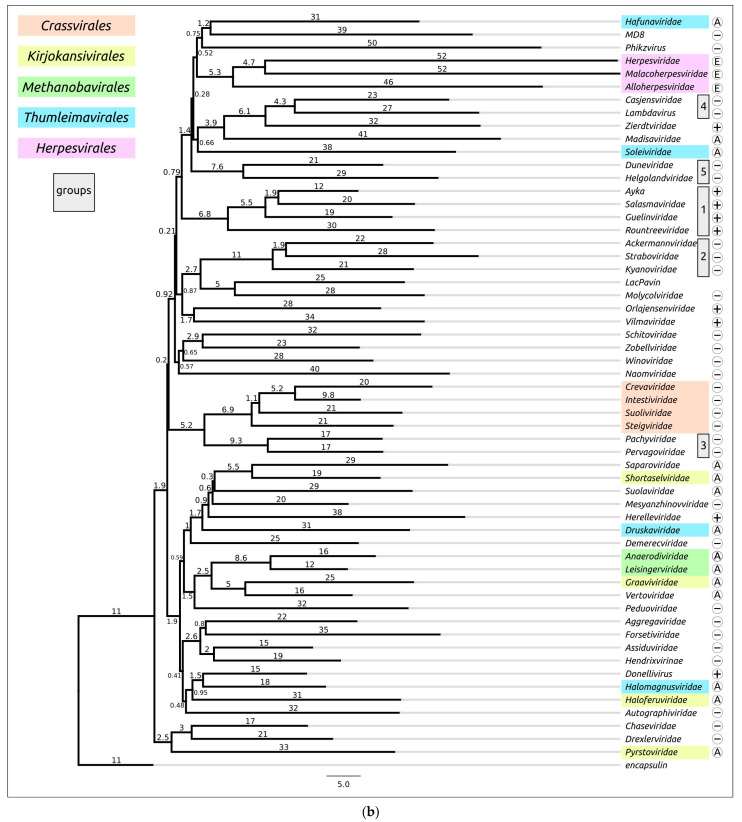
Heatmap (**a**) and dendrogram (**b**) based on the pairwise Z-score comparisons of 57 major capsid proteins and encapsulin AF models, using DALI. The branch lengths are measured using the DALI Z-score, and the tree was rooted to encapsulin. “A”—archaeal viruses, “E”—eukaryotic viruses, “+”—phages infecting Gram-positive bacteria, and “−”—phages infecting Gram-negative bacteria. Groups correspond to clusters found as a result of structural comparison. Reprinted/adapted with permission from Ref. [55]. © 2023 by the authors.

Structural similarity is widely used to evaluate evolutionary relationships between proteins whose amino acid sequence homology level is low or cannot be determined at all [104,105]. The structural similarity between two proteins can be assessed using root-mean-square deviation (RMSD) or other metrics such as template modelling score (TM-score) and DALI Z-score; the latter two metrics have a number of advantages over RMSD [84,106]. Clustering of experimentally determined structures of major capsid proteins using the DALI Z-score has already been used to illustrate the common origin of some viral groups and to cluster prokaryotic viruses [56,104]. Integrated use of both experimental structures and AF2-derived structures can be used for elucidation of evolutionary relationships and taxonomic classification of bacteriophages and eukaryotic viruses [57,59,107]. AlphaFold modelling and subsequent clustering have been used in taxonomic studies of archaeal viruses [56]. Clustering using AlphaFold showed interesting and often biologically meaningful results [55]. Clustering using structures predicted by AlphaFold showed interesting and often biologically meaningful results (Figure 7). It should also be noted that the native state of viral proteins can change according to the state of the viral particle (e.g., empty, full, expanded capsids) and according to the stage of viral particle assembly [108,109,110,111]. The correlation between structural similarity and sequence identity is not absolute due to conformational plasticity, solvent effects and ligand binding [112]. Most of these limitations apply to studies that involve experimentally determined structures, but, hypothetically, they could be exacerbated by structural prediction errors. Therefore, predicting the effectiveness of using AlphaFold for the analysis of structural similarity and evolutionary history, based only on the similarity of the predicted structures, seems to be a difficult task [55].

## 5. Further Development of AlphaFold and Machine Learning Techniques

### 5.1. AlphaFold-Multimer and Prediction of Multi-Chain Protein Complexes

Originally, AF2 was designed to predict monomeric protein structures. Consequently, interactions between different proteins, subunits and domains in multimers were not described in the AlphaFold database [61]. As a result, some large multi-domain protein complexes may not have been modelled accurately enough. Several publications have, however, explored how AF2 could be used for predicting both homo- and heteromeric complexes [62,63,64]. Moreover, it has been pointed out that an AI system outperforms standard docking methods in as much as it does not require starting protein structures [62].

In addition, a number of approaches have been developed to make AlphaFold work well for complicated protein structures with multiple bindings. Recent versions of AF2, such as those incorporated into ColabFold, enable multimer structures to be uploaded [63]. They include AlphaFold-Multimer, the extension developed by the DeepMind team, which significantly improves the accuracy of predicting multimeric interactions [19]. This new instrument is an AlphaFold algorithm that is specially modified to use multimeric data and trained on oligomeric proteins. However, there is evidence that this multimeric modification has not succeeded in predicting the key features of some protein complexes [65]. Currently, AlphaFold-Multimer does not include the self-distillation of multimer predictions, so the authors believe there is potential for future accuracy enhancements.

To overcome the limitation described above, combining AF2 with experimental methods, e.g., cryo-electron tomography and/or other computer-based tools such as RoseTTAFold, provides more robust results [64,66]. Other authors have suggested combining AlphaFold models of protein complexes with differential covalent labelling mass spectrometry data by applying RosettaDock [67]. The use of cryo-electron microscopy maps, integrated with AlphaFold, for multi-chain protein complex prediction also encourages the creation of accurate and reliable models [68].

Other approaches include the use of optimised multiple sequence alignment together with AF2 [69] and the application of a Monte Carlo tree search [70]. The latter works well but only with symmetric protein complexes and when the stoichiometry of the subcomponents is known.

### 5.2. AlphaFill

A study from Massachusetts Institute of Technology, which mainly focused on the limitations of AF2 in the drug industry [74], showed that the use of AF2 together with molecular docking simulations to predict protein-ligand bindings demonstrated poor performance that, in some cases, was comparable to pure chance. At the same time, this study indicated how prediction accuracy might be improved with the integration of machine-learning-based approaches. The authors of the study expected their research to encourage the development of machine-learning methods that would complement AlphaFold.

AlphaFill is a new tool that has been developed to solve the problem with ligands and cofactors in the AlphaFold protein structure database [75]. AlphaFill uses an algorithm that employs sequence and structure similarity analysis to graft missing molecules and ions from experimental data into predicted protein structures. The algorithm has been successfully validated against experimental structures.

## 6. Critique of AlphaFold

AlphaFold has probably revolutionised the determination of protein molecular structure. Today, AF2 is a state-of-the-art deep-learning tool that demonstrates an accuracy in predicting protein folding that was previously unattainable using computational tools. The quality of its predictions is, however, not consistent. Furthermore, in some cases, Artificial Intelligence (AI) systems are unable to provide highly accurate results. As reported by the EMBL’s European Bioinformatics Institute, 35% of the more than 214 million AF2 predictions have been found to be very accurate [60], which indicates that its predictions are often not inferior to those obtained experimentally. It should also be pointed out that 45% of these predictions still could be used for some applications, in spite of their accuracy being inferior to that of experimentally retrieved structures. Therefore, although AF2 is an outstanding tool, it is important to consider its limitations to ensure that investigations provide reliable results.

### 6.1. Intrinsically Disordered Proteins and Intrinsically Disordered Protein Regions

When AlphaFold encounters difficulties with obtaining highly accurate predictions, the problem very often relates to intrinsically disordered proteins (IDPs) or intrinsically disordered protein regions (IDRs) [71]. AI systems perform excellently when predicting well-folded proteins, but about a third of eukaryotic proteins are intrinsically disordered or contain disordered regions [72]. Moreover, IDPs play an important role in physiological functions, such as in protein signalling networks.

The reason for AlphaFold encountering difficulties when predicting IDRs may be that these proteins and regions are often not solved by X-ray crystallography; AF2 is mainly designed to use X-ray data [62]. There is a database, DisProt, that contains consolidated information on IDPs [72]. If AF2 or another AI system could be tailored so that it can extract conformational features from DisProt or some other experiment-based databases, then this might enable prediction of IDPs/IDRs in the future.

### 6.2. Protein Interactions with Metal Ions, DNA, RNA, Cofactors, Ligands and Post-Translational Modifications

Many proteins can physiologically function only in the form of complexes with various ions and molecules, such as hemoglobin. Such interactions are especially crucial for drug discovery. It is to be expected, therefore, that much of AlphaFold’s criticism is related to the fact that it omits protein-ligand interactions in its predictions [18,73].

AlphaFold is not designed for the prediction of post-translational modifications (PTMs) of proteins, such as protein glycosylation. This fact has attracted the attention of the scientific community, with recent studies demonstrating the relevance and importance of glycosylation in the SARS-CoV-2 spike protein or in human proteins. According to research, between 50% and 70% of the 20,000 predicted human proteins are thought to be glycosylated [113]. Bagdonas et al. suggested that the use of sequence- and structure-based studies might address not only the ligand and cofactor interactions problem but also issues related to PTMs [76]. The authors presented an example of glycosylation to demonstrate the potential of their proposed approach, developing an algorithm integrated into Privateer software. This tool ‘transfers’ protein glycosylation from a library of structurally balanced glycan blocks to the protein folding from AlphaFold.

### 6.3. Protein Conformations

Proteins are not static; they take on various structures, depending on their surroundings or the stage in the functional cycle. Conformational changes in proteins are closely related to their functions and regulations. They can be caused by binding to other molecules, by PTMs or by changes at the pH and temperature levels, for example. AlphaFold provides a static picture of protein folding and does not incorporate information about its dynamics [77]. There is also no clarity as to which conformation of the protein will be predicted by AlphaFold [61]. Consequently, this AI system offers only partial information about the key features of the relationship between protein structure and function.

The situation is complicated by the fact that data on these conformations obtained under experimental conditions also have limitations. Nevertheless, at the moment, it seems that predictions of conformations and the dynamics of protein structures are only possible using experimental methods, such as time-resolved crystallography and structural distributions from cryo-EM data [78].

### 6.4. Mutations

According to some studies, it appears that AF2 is unable to predict defects in protein structures caused by mutations [79]. One investigation showed that differences between mutated and wild-type structures predicted by AlphaFold were extremely small [80]. Other researchers have found that it is impossible to obtain a reliable evaluation of the impact of mutation on protein stability with the direct application of AI predictions [81]. Thus, predicting the effect of mutations on protein stability should be carried out as a specific task, although this will be hampered by the limited amount of data available for training deep-learning models.

### 6.5. Database Loopholes

As a deep neural network, AF2 cannot correctly predict absolutely unknown structures on which it was not trained. It is based on MSA and experimentally obtained structures stored in the database. Similarly, AF2 also lacks predictive accuracy where fewer sequences are available for alignment [65]. Accordingly, the AI’s quality performance will depend on how much experimental and previous computational data have been collected and stored in databases. This is not really a limitation, since it may be considered as an opportunity, given that the more data that are collected, the more accurate predictions will become.

## 7. Conclusions

Protein structure modelling is an important task that helps fundamental and applied research in the field of virology. The AlphaFold deep-learning algorithm, which has been proven to be a highly accurate prediction method, can be used in the design of new drugs and in studies of viral pathogens and mechanisms of viral infection. In bacteriophage research, AlphaFold predictions can also be used to model receptor-binding proteins and glycopolymer-degrading enzymes, helping to develop new antibacterials and biocontrol agents.

## Figures and Tables

**Table 1:** Summary of the studies discussed.

Authors, Year	Virus or Viral Group	Study Aim (s)	Results and AF2 Usage
Callaway 2022 [18]		to explore how AF2 changes biology	AF2 affects many studies and provides a quality of prediction not previously achievable by computational tools. At the same time, it has limitations and it is important to consider them when conducting research.
Evans et al., 2021 [19]		to present the extension of AlphaFold for protein complexes—AlphaFold-Multimer	AlphaFold-Multimer significantly improves the quality of predicted multimeric interfaces, compared with basic AlphaFold adapted to input data, while maintaining a high level of accuracy within the chain.
Abdelkader et al., 2022 [25]	SARS-CoV-2	to find inhibitors of non-structural protein 6 (NSP6)	Using the AF2 predictions, candidate inhibitors were suggested and recommended for biological testing.
Flower et al., 2021 [26]	SARS-CoV-2	to test the in silico prediction of β-rich ORF8 protein for finding an MR solution to the crystallographic phase problem	It was shown that the ORF8 protein model, predicted by AF2, is sufficiently accurate to provide a phase solution by MR.
Vuren et al., 2022 [27]	SARS-CoV-2	to test highly thermo-tolerant monomeric receptor-binding domain derivatives on mice for the development of new vaccines	The monomeric formulation of the vaccine was observed to produce a slightly superior immune response, possibly because it presents more antigenic epitopes, as shown using AF2 predictions.
Singanallur et al., 2022 [28]	SARS-CoV-2	to assess leading vaccines in virus neutralisation assays against Delta and Omicron variants of concern (VOC) and a reference isolate	At least a third dose of these vaccines is necessary to generate sufficient neutralising antibodies against emerging VOC. AF2 was used to find an explanation for the observed reduction in neutralisation of Omicron compared with other variants.
Bhowmick et al., 2022 [29]	SARS-CoV-2	to study the effects of various mutations in the RBD of the SARS-CoV-2 spike and its key interactions with the ACE-2 receptor, using protein structure prediction algorithms along with molecular docking	AF2-generated and trRosetta-generated models of RBD were compared. trRosetta predictions appeared to be more accurate and have been used for docking with the ACE-2 receptor of other mutated RBD variants.
Robertson et al., 2021 [30]	SARS-CoV-2	to evaluate the concordance of AF2 models with residual dipolar couplings data	Close agreement between all sets of AlphaFold models and experimental residual dipolar couplings data was found for most of the protein.
Kumari et al., 2023 [31]	Monkeypox virus (MPXV)	to search for inhibitors of MPXV DNA polymerase (DNAP) for antiviral therapy	DNAP inhibitors were found using an AF2-generated model and virtual screening of ZINC and antiviral libraries.
Kannan et al., 2022 [32]	MPXV	to study the effects of mutations in DNA replication complex (RC)	Mutations in RC that are likely to contribute to the 2022 monkeypox outbreak were identified. AF2 predictions were used to model an RC component.
Li et al., 2022 [33]	MPXV	to study the mechanisms of inhibition of poxvirus phospholipase D (F13) by tecovirimat, which have been demonstrated to be effective against monkeypox in vitro and in anima	The potential binding pocket and the possible binding mode for tecovirimat with F13 were revealed using AF2 structure predictions and molecular docking.
Yefet et al., 2023 [34]	MPXV	to characterise the main serological and B cell markers accompanying MPXV infection in humans	The reactivity of three MPXV antigens to MPXV-11convalescent sera and responses caused by vaccinia virus-based vaccine (VACV) were tested. AF2 modelling indicated similar conformations of MPXV and VACV antigens.
Benedyk et al., 2022 [35]	Herpes Simplex Virus Type-1 (HSV-1)	to study the mechanisms of influence of HSV-1 on sphingolipid metabolism	Using AF2 predictions, the residues essential for the binding of involved proteins were identified and experiments demonstrating that HSV-1 modifies the sphingolipid metabolism via specific protein–protein interactions were conducted.
Collantes et al., 2022 [36]	HSV-1	to study details of the transport of the viral particle towards the nucleus	Structural features of the UL37 tegument protein, which is important for retrograde transport and viral replication, were revealed. AF2 and other computational techniques were used for prediction of structures of UL37 and binding surface.
Fieulaine et al., 2023 [37]	Hepatitis E virus (HEV)	to study HEV replication polyprotein (pORF1)	The structure of HEV pORF1 was obtained with AF2 and then analysed. The protocol to express and purify the full-length HEV pORF1 was developed.
Liu et al., 2022 [38]	Rice black-streaked dwarf virus (RBSDV)	to reveal lipid-binding sites of major outer capsid protein (also known as P10)	The use of AF2 predictions and the results of experimental studies enabled the suggestion of putative binding sites of lipids on RBSDV P10 protein.
Chen et al., 2022 [39]	African swine fever virus (ASFV)	to study the mechanism of interactions of ssDNA and ssDNA-binding protein CP312R	With the assistance of AF2 predictions, the crystal structure of ASFV CP312R was determined, and the putative ssDNA binding core domain was suggested.
Kim et al., 2023 [40]	Viral hemorrhagic septicemia virus (VHSV)	to study the genesis of secondary mutations in the matrix (M) protein	VHSV was found to respond to the artificial mutation of M protein through secondary mutations. These secondary mutations occurred when the artificial mutations were harmful for the virus. AlphaFold was used to predict the structure of the M protein.
Veit et al., 2022 [41]	Porcine reproductive and respiratory syndrome virus (PRRSV)	to study the Gp5/M protein dimer, the major component of the viral envelope required for virus budding	Detailed bioinformatic analysis of Gp5/M was conducted using various bioinformatic tools. AlphaFold was used to obtain a model of the Gp5/M dimer.
Hötzel 2022 [42]	Several lentiviruses and betaretroviruses	to study the surface envelope glycoproteins of nonprimate lentiviruses and betaretroviruses	The consistence of AF2 models of small ruminant lentiviruses and betaretroviruses and experimental data was shown. Structural features of gp135 of small ruminant lentiviruses were discussed.
Weaver et al., 2022 [43]	Human roseolovirus	to clarify structural features of membrane glycoproteins U20 and U21	AlphaFold and RoseTTAfold were used to predict the structures of U20 and U21. Structural features of these proteins were discussed.
Al-Shayeb et al., 2022 [44]	Bacteriophage metagenomic sequences	to study CRISPR systems encoded in phage genomes	Bacteriophage-encoded CRISPR systems were found and classified using genome-resolved metagenomics. The Casλ-RNA-DNA structure was determined using Cryo-EM. AF2 was used to obtain the initial model of the Casλ protein.
Klumpp et al., 2023 [45]	Various bacteriophages	to review the features and use of phage receptor-binding proteins (RBPs)	Distinctive features of phage RBPs, the use of RBPs as antibacterial agents and the application of AlphaFold for the prediction of RBPs’ structure were described.
Goulet et al., 2021 [46]	*Oenococcus oeni* phages OE33PA and Vinitor162	to reveal the structural features of different phage adhesion devices	The topology and structure of phage adhesion proteins was studied using AF2 modelling. Based on known models, a topological model of the OE33PA adhesion device was proposed.
Evseev et al., 2023 [47]	*Curtobacterium* prophages	to reveal and characterise *Curtobacterium* prophage-derived regions and glycopolymer-degrading enzymes of prophage origin	Prophage-derived regions were found and annotated. Glycopolymer-degrading enzymes of prophage origin were modelled using AF2, characterised and clustered.
Hawkins et al., 2022 [48]	*Staphylococcus* phage Andhra	to study the phage’s structural features	The Cryo-EM structure was reported. Using AlphaFold predictions, the distal tail model was built.
Nieweglowska et al., 2023 [49]	*Pseudomonas* phage ϕPA3	to explore the mechanism of formation of the phage nucleus	The ability of Phage Nuclear Enclosure (PhuN) protein to spontaneously assemble into 2D sheets with p2 and p4 symmetry was shown. The p2 symmetric state was resolved by Cryo-EM. AF2 was used to build a model of the 2D array.
Šiborová et al., 2022 [50]	*Escherichia* phage SU10	to study the mechanism of phage genome delivery	Cryo-EM and Cryo-ET characterisation of the attachment of the phage to the host cell was presented. The formation of a tail nozzle after rearrangement was shown. AF2 was used to build tail models.
Conners et al., 2021 [51]	*Klebsiella* phage f1	to study the structural bases of the mechanism of phage egress and its practical application	Cryo-EM structure phage-encoded pIV secretin was determined, and the mechanism for phage egress was proposed. AF2 was used to predict the structure of the N0 domain of pIV.
Eskenazi et al., 2022 [52]	*Klebsiella* phage M1	to investigate the effectiveness of combined pre-adapted bacteriophage therapy and antibiotics for the treatment of fracture-related infection	The therapy resulted in an objective improvement in the patient’s wounds and overall condition. The combination of phage and antibiotic therapy was demonstrated to be highly effective against the patient’s *K. pneumoniae* strain. AlphaFold was used for the modelling of original and mutated phage proteins.
McGinnis et al., 2022 [53]	*Mycobacterium* phage TipsytheTRex	to study the mechanism of the interaction of the immunity repressor and DNA	A Dual DNA binding domains model of the repressor was proposed. An AlphaFold model of the repressor protein was used to significantly improve the structure obtained using single-wavelength anomalous diffraction phasing.
Zhang et al., 2022 [54]		to investigate the functioning of the toxin–antitoxin system CapRel^SJ46^ that protects *E. coli* against phages	It was shown that the C-terminal domain of CapRel^SJ46^ controls the toxic N-terminal region. Major capsid proteins of some phages bind to the C-terminal domain to relieve autoinhibition, enabling the toxin domain. AF2 was used for predicting different conformations of CapRel^SJ46^.
Evseev et al., 2023 [55]	Various archaeal and bacterial *Duplodnaviria* viruses	to clarify the classification of high-ranked taxa	Using the results of AlphaFold predictions, combined with the results of sequence-based phylogeny, suggestions for possible upgrades to taxonomic classifications of *Duplodnaviria* viruses were made.
Liu et al., 2021 [56]	Archaeal tailed viruses	to study and classify archaeal tailed viruses, including newly sequenced ones	A total of 37 newly sequenced genomes and published sequences were classified using genomic similarity and network-based analysis. AF2 was used for modelling major capsid proteins and further structural comparisons.
Podgorski et al., 2023 [57]	Actinobacteriophages	to classify actinobacteriophage major capsid proteins	AlphaFold predictions, together with experimentally obtained structures, were used to construct a detailed structural dendrogram describing the evolution of capsid structural stability within actinobacteriophages.
Evseev et al., 2022 [58]	Various myoviruses	to reveal patterns of structural evolution of tail sheath protein	Based on AF2 predictions and laboratory-derived structures, patterns of evolution of phage sheath protein were revealed.
Hötzel et al., 2022 [59]	Various retroviruses	to clarify common structural features of the retroviral surface envelope protein subunit (SU)	Analysis of structures predicted with AF2 revealed the common conserved core of Sus and enabled the identification of a homologue structure in the SU equivalent GP_1_ of filoviruses, demonstrating their common origin.
Callaway 2022 [60]		to present the results of the first year of AF2	The AlphaFold tool predicted about 200 million protein structures. About 35% of these structures were highly accurate and 45% could be used for specific purposes.
Perrakis et al., 2021 [61]		to consider the scope and implications of AF2 applications in structural biology	Despite a number of limitations, the analysis of models obtained with AlphaFold can generate new and testable hypotheses about protein function, which is necessary for structural biology.
Akdel et al., 2022 [62]		to evaluate the use of AF2 predictions for different structural biology challenges, such as variant effect prediction, pocket detection, and model	AF2 predictions, given their limitations, can be applied to existing structural biology problems, and their accuracy is close to that of experimental models.
Mirdita et al., 2022 [63]		to present ColabFold and its comparison with other tools	ColabFold goes beyond the original AF2 functions by improving sequence searches, providing tools for modelling protein complexes, extending databases and determining protein structures, with about 90 times the speed of AF2.
Humphreys et al., 2021 [64]		to obtain models for 106 previously unidentified protein complexes and 806 proteins, for which detailed structural information was lacking	The combination of AlphaFold and Ro-seTTAfold expanded the scope of deep-learning-based tools for modelling protein complexes.
Gomes et al., 2022 [65]		to assess the reliability of AlphaFold predictions of Staphylococcus bacteria adhesins proteins, using single-molecule force spectroscopy	AlphaFold generates extremely robust protein structures, but in some cases cannot accurately predict protein multimers. Even AlphaFold Multimer failed to predict important structural features for some of the investigated complexes, such as the locking strand of adhesin.
Subramaniam et al., 2022 [66]		to study a combination of computational and experimental tools for protein structure prediction	It was concluded that the development of structural biology in the future will be closely related to the synergy between deep-machine-learning-based predictions, as in AF2, and cryo-EM technology.
Drake et al., 2022 [67]		to propose a new hybrid method of Alphafold, Rosetta and mass spectrometry covalent labelling for predicting protein complexes	Combining AF2 models of protein complexes with differential covalent labelling mass spectrometry data via the application of RosettaDock demonstrated a lower root-mean-square deviation than complexes predicted without covalent labelling data.
He et al., 2022 [68]		to present EMBuild, an automatic model-building tool for protein complexes	EMBuild automatically builds models from intermediate-resolution cryo-EM maps integrating AlphaFold structure prediction. It provides quality and reliable models that are comparable to manually built structures.
Bryant et al., 2022 [69]		to offer a new protocol for AF2 prediction of protein complexes	The use of optimised multiple sequence alignment together with AF2 showed acceptable quality for 63% of the dimers.
Bryant et al., 2022 [70]		to propose the use of Monte Carlo tree search for predicting protein complexes with AF2	The application of a Monte Carlo tree search for the predicted AF2 subcomponents yielded 91 of 175 complexes, with a median TM-score of 0.51, and 30 of them demonstrated high accuracy.
Ruff et al., 2021 [71]		to study the implications of AlphaFold for intrinsically disordered proteins	Predicted structures obtained with AlphaFold emphasised the importance of intrinsically disordered proteins/regions. A huge number of protein regions that AlphaFold predicted with low accuracy overlapped with regions predicted as IDRs.
Laurents et al., 2022 [72]		to provide information on the prediction of protein folding using a combination of NMR and AF2 spectroscopy	In the future, NMR spectroscopy may strengthen Alphafold predictions in areas where it has limitations: conformations, ligand and cofactor interactions, post-translational modifications and intrinsically disordered proteins.
Edich et al., 2022 [73]		to study the impact of AF2 on experimental structure solution	Although AF2 has some drawbacks, it can help in the design of the experiment and determine which part of the protein sequence may be intrinsically disordered. It also encourages the conducting of more experimental studies, as data from them can improve deep-machine-learning’s ability to predict.
Wong et al., 2022 [74]		to assess AlphaFold-enabled molecular docking predictions for drug discovery	The use of AF2 together with molecular docking simulations to predict protein-ligand bindings demonstrated poor performance. The prediction accuracy might be improved by the integration of machine-learning-based approaches.
Hekkelman et al., 2022 [75]		to present AlphaFill, a tool for improving AlphaFold predictions with ligands and cofactors	The developed algorithm, employing sequence and structure similarity analysis, received a good validation performed against experimental structures.
Bagdonas et al., 2021 [76]		to propose an approach that addresses the absence of cofactors and co- or post-translational modifications in AF2 models	This approach combines sequence and structure data to transfer protein glycosylation from a library of structurally balanced glycan blocks to the AlphaFold model. The algorithm was integrated into the Privateer software.
Van Breugel et al., 2022 [77]		to assess the quality of AF2 models in the study of centrosome and centriole biogenesis	AF2 models can reveal important insights into the structural features of two key proteins in centrosome and centriole biogenesis, CEP192 and CEP44. The AF2 algorithm was used to predict, with subsequent experimental validation, previously unknown primary features in the structure of TTBK2 associated with CEP164, as well as the Chibby1-FAM92A complex.
Lane 2023 [78]		to discuss AF2 restrictions concerning structural distribution and other issues	As deep-machine-learning algorithms develop, they require more and more experimental data. In the author’s opinion, experimental methods such as time-resolved crystallography, cryo-EM data and others can provide information that enables researchers to penetrate the essence of protein functioning.
Bertoline et al., 2023 [79]		to provide an overview of changes in protein structure prediction before and after the advent of AF2	The advent of AF2 has taken the protein folding prediction problem to the next step; however, it has several limitations. AF2 instigated the emergence of new tools, such as ESMfold, which, although inferior in accuracy, use different approaches, which enable very fast predictions.
Buel et al., 2022 [80]		to study the ability of AF2 to predict the effect of missense mutations on structure	AF2 seems not to be able to predict the effect of missense mutations on the 3D structure of proteins. Differences between mutated and wild-type structures predicted by AlphaFold were extremely small.
Pak et al., 2021 [81]		to evaluate the ability of AlphaFold to predict the impact of single mutations on protein stability	It seems impossible to obtain a reliable evaluation of the impact of mutation on protein stability with the direct application of AI predictions.

## Data Availability

Not applicable.
